# Ovarian cancer histology-specific incidence trends in Canada 1969–1993: age-period-cohort analyses

**DOI:** 10.1038/sj.bjc.6690665

**Published:** 1999-09

**Authors:** J Zhang, A-M Ugnat, K Clarke, Y Mao

**Affiliations:** Environmental Risk Assessment and Case Surveillance Division, Laboratory Centre for Disease Control, AL 0601C1, Health Canada, Ottawa, K1A 0L2, Canada

**Keywords:** ovarian cancer, incidence, histology, period effect, cohort effect, age-period-cohort analysis

## Abstract

This study examined histology-specific incidence trends of ovarian cancer in Canada, 1969–1993. The impact of age, period and cohort effects on these trends were studied by means of age-period-cohort analysis. Age-standardized incidence rates of serous, endometrioid, clear cell and germ cell tumours increased significantly and the rates of sex cord-stromal and other classified epithelial ovarian tumours decreased considerably. The rates of mucinous and NOS/unclassified tumours remained unchanged. Cohort effect has a major impact on incidence trends of serous, endometrioid, germ cell, sex cord-stromal and other classified epithelial ovarian tumours but no meaningful impact on trends of mucinous, clear cell, or NOS/unclassified ovarian tumours. Various cohort patterns by histology subtypes were observed: the risk of developing serious tumours increased markedly among birth cohorts of 1895–1930, stabilized thereafter and decreased among young cohorts of 1950–1960; the risk of germ cell tumours increased significantly among young cohorts of 1965–1980; and the risk of sex cord-stromal tumours dropped constantly among cohorts 1910–1950. Various period patterns by histology subtypes observed in this study suggested changes in histology classification criteria over the period. Further studies need to consider the various etiologies and the classification criteria changes according to histology subtypes. © 1999 Cancer Research Campaign


*British Journal of Cancer* (1999) **81**(1), 152–158
© 1999 Cancer Research Campaign
Article no. bjoc.1999.0665

### Ovarian cancer histology-specific incidence trends in Canada 1969–1993: age-period-cohort analyses

**J Zhang, A-M Ugnat, K Clarke and Y Mao**

Environmental Risk Assessment and Case Surveillance Division, Laboratory Centre for Disease Control, AL 0601C1, Health Canada, Tunney's Pasture, Ottawa, Ontario, Canada K1A 0L2

**Summary** This study examined histology-specific incidence trends of ovarian cancer in Canada, 1969–1993. The impact of age, period and cohort effects on these trends were studied by means of age-period-cohort analysis. Age-standardized incidence rates of serous, endometrioid, clear cell and germ cell tumours increased significantly and the rates of sex cord-stromal and other classified epithelial ovarian tumours decreased considerably. The rates of mucinous and NOS/unclassified tumours remained unchanged. Cohort effect has a major impact on incidence trends of serous, endometrioid, germ cell, sex cord-stromal and other classified epithelial ovarian tumours but no meaningful impact on trends of mucinous, clear cell, or NOS/unclassified ovarian tumours. Various cohort patterns by histology subtypes were observed: the risk of developing serious tumours increased markedly among birth cohorts of 1895–1930, stabilized thereafter and decreased among young cohorts of 1950–1960; the risk of germ cell tumours increased significantly among young cohorts of 1965–1980; and the risk of sex cord-stromal tumours dropped constantly among cohorts 1910–1950. Various period patterns by histology subtypes observed in this study suggested changes in histology classification criteria over the period. Further studies need to consider the various etiologies and the classification criteria changes according to histology subtypes.

**Keywords:** ovarian cancer; incidence; histology; period effect; cohort effect; age-period-cohort analysis

Ovarian cancer is the fourth most common cancer diagnosed among Canadian women. Approximately 2500 women (14.0 per 100 000) will be diagnosed with ovarian cancer and 1400 will die from this disease in 1998 (National Cancer Institute of Canada, 1998). In Canada, the incidence rate of ovarian cancer has declined slightly over the past two decades; however, age-specific incidence rates show different patterns (On and Semenciw, 1995).

The aetiologies of ovarian cancers, especially of non-epithelial ovarian tumours (germ cell and sex cord-stromal) are poorly understood. Previous epidemiological studies have focused on aetiologies of epithelial tumours (serous, mucinous, endometrioid, clear cell, etc.) (Whittemore et al, 1992). Several factors including advanced age (Ewertz and Kjaer, 1988), nulliparity (Mosgaard et al, 1997) and a family history of ovarian cancer (Amos and Struewing, 1993) have been consistently associated with an increased risk of epithelial ovarian cancer, while other factors such as number of full-term pregnancies (Risch et al, 1994*a*), oral contraceptive (OC) use (Rosenberg et al, 1994) and history of hysterectomy or tubal ligation (Hankinson et al, 1993) have been consistently associated with a decreased risk. Evidence regarding the roles of other factors, including age at menarche/menopause and age at first childbirth (Negri et al, 1991), hormone replacement therapy (HRT) (Hempling et al, 1997), infertility and fertility drug use (Risch et al, 1994*a*), breastfeeding (Siskind et al, 1997), obesity (Farrow et al, 1989), diet (Risch et al, 1994*b*), talc use (Chang and Risch, 1997), smoking and alcohol or coffee consumption (Whittemore et al, 1988), is inconclusive.

On the other hand, few studies have focused on aetiologies on non-epithelial ovarian tumours. Walker et al (1988) observed an elevated risk of germ cell ovarian cancer among girls and young women, the mothers of whom were under 20 years of age at time of pregnancy, had used exogenous hormones during the pregnancy or had a high pre-pregnancy body mass. Use of oral contraceptives was associated with an increased risk of germ cell tumours (Horn-Ross et al, 1992). However, a history of oral contraceptives use or oestrogen replacement therapy was associated with a decreased risk of developing sex cord-stromal ovarian tumours.

Cohort effects are reported to have an important impact on ovarian cancer incidence time trends. An earlier study in Denmark reports a remarkable increasing risk of ovarian cancer for cohorts of women born between 1863 and 1898 and the risk stabilized for cohorts born thereafter (Ewertz and Kjaer, 1988). Significant cohort effects on ovarian cancer incidence trends have also been observed in Sweden, England/Wales and Norway (Adami et al, 1990; Dos Santos Silva and Swerdlow, 1995; Bjorge et al, 1997). These studies generally found that the risk of developing ovarian cancer increased sharply among women born during the last decades of the nineteenth century, and the risk increased slowly among women born during the first decades of the twentieth century and declined for cohorts born in recent decades.

Although reports on incidence trends are available for ovarian cancer overall, reports on histology-specific trends are sparse (Cramer et al, 1981; Farrow and Rosenblatt, 1996). The purpose of this study is to investigate ovarian cancer incidence trends

*Received 14 September 1998*
*Revised 18 January 1999*
*Accepted 9 February 1999*

*Correspondence to:* Y Mao according to histology subtype and to study the impact of age, period and cohort effects on these trends in Canada during the time period 1969–1993.

### MATERIALS AND METHODS

### Data source

Ovarian cancer incidence data including histology-specific data were obtained from provincial cancer registries through the Canadian Cancer Registry (CCR) at Statistics Canada for the study period 1969 to 1993. Annual population estimates were obtained from the Demography Division of Statistics Canada for the same period. Since histology-specific data were available for the full study period only for provinces of British Columbia, Ontario and Saskatchewan, this study was based on data obtained from these three provinces only.

British Columbia and Saskatchewan were the first two provinces in Canada to have cancer registries (Health and Welfare Canada, 1993); by 1935 the two provinces had established population-based cancer registries. By 1965 Ontario had also built a population-based cancer registry. The provincial cancer registries obtain cancer incidence information from various sources including patient diagnosis and treatment records, pathology reports, hospital separation records, death certificates and autopsy reports. In these three provinces, the proportion of microscopically confirmed ovarian tumours remained 87–90% and the proportion of cases identified by death certificate only (DCO) stayed 2–3% during the study period.

Ovarian cancer, defined as a malignant neoplasm of the ovary, was coded as 183.0 in the 8th (1969–1978) and 9th (1979–1993) revisions of the International Classification of Diseases (ICD-8, ICD-9) (WHO, 1977). 'Borderline' ovarian tumours were excluded in the analysis. Histology subtypes were identified using the International Classification of Diseases for Oncology (ICDO) (WHO, 1976; Berg, 1982). Major subtypes included serous tumours (ICDO 8441, 8442, 8460, 8461, 8462), mucinous tumours (ICDO 8470, 8471, 8472, 8473, 8480, 8481), endometrioid tumours (ICDO 8380, 8381, 8560), clear cell tumours (ICDO 8310), germ cell tumours (ICDO 906–910), sex cord-stromal (ICDO 859–867), all other classified epithelial ovarian tumours (ICDO 8010, 8021, 8050, 8140, 8260, 8440, 8450, 8570, 8950, 8951, 8980, 9110, etc.) and NOS (not otherwise specified)/unclassified tumours (ICDO 800 and 999).

### Statistical analysis

Annual age-standardized incidence rates (ASIRs per 100 000 women) for ovarian cancer and its histology subtypes were calculated for 1969–1993 by the direct method, using the 1991 Canadian census population as standard. Histology-specific temporal trends of the ASIRs were studied using average annual per cent change (AAPC), approximated with logarithm regression coefficients and tested by *t*-tests (National Cancer Institute of Canada, 1998).

Age-specific incidence rates (per 100 000 women) for each histology subtype were calculated according to age group (0–4, …, 80–84, 85+) for the period 1989–1993.

To study the impact of calendar period and birth cohort on histology-specific incidence trends of ovarian cancer, log-linear regression models were applied assuming that the observed number of cancer cases followed a Poisson distribution. Several models were used: full model (APC) including three factors, age (A), calendar period (P) and birth cohort (C); two-factor models consisting of age and period (AP) or age and cohort (AC); and one-factor models including age (A) or age-drift (AD) in which the period is treated as a continuous variable (Clayton and Schifflers, 1987*a*, 1987*b*). The SAS procedure GENMOD (SAS Institute Inc., 1996) was used to compute parameter estimates for age, period and cohort.

For each histology-specific subtype, various models including A, AD, AP, AC and APC were fitted. Goodness-of-fit of the various models and the overall significance of age, cohort, or period effect were tested using log-likelihood test by means of the scaled deviance. A suggested best model was determined for each of the histology subtypes based on model fit. The best model indicates the best statistical approach to describe the impact of age, period and cohort effects on a specific incidence time trend. For example, the best model APC means that age, period, cohort are all significantly associated with the incidence trend, while AC indicates that only age and cohort are significantly related to the incidence trend. Parameter estimates were computed through the best model and the significance of these estimates was tested using the $\chi^2$ test.

Due to the linear relationship between age, period and cohort, parameter estimates of birth cohorts cannot be unequivocally identified and a constraint must be applied. In this study, the parameters were estimated under the constraint that the effects for the first and the last birth cohorts were equal to zero. Since different constraints may result in different sets of parameter estimates, the magnitude of the parameter estimates cannot be interpreted using relative risk (RR) and 95% confidence interval (CI). However, remarkable changes in the direction of birth cohort or time period effects remain the same under different constraints (Tarone and Chu, 1996). In general, these direction changes may suggest meaningful impacts of period and/or cohort effects on incidence trends. For example, a notable change in the direction of birth cohort effect may suggest an increasing or decreasing exposure to a risk factor among that birth cohort. A sharp change in the direction of period effect may indicate an improved data capture process in cancer registries, an enhanced cancer diagnosis method, introduction of an efficient screening test, or changes to a more recent histological classification.

For analyses of overall ovarian cancer and all subtypes except germ cell tumours, the data were organized into twelve 5-year age groups (25–29, 30–34, …, 80–84), five 5-year time periods (1969–1973, …, 1989–1993) and seventeen overlapping 10-year birth cohorts (1884–1993, 1889–1998, …, 1959–1968). Each birth cohort was identified by the 7th year of the interval (i.e. 1890, 1895, …, 1965). For example, the 1895 birth cohort represents women born from 1889 through 1898, about 50% of these born between 1891 and 1895. Note that the 1895 birth cohort overlaps both with the 1890 and the 1900 birth cohort by 25%. For analysis of germ cell tumours, the data were organized into fourteen 5-year age groups (0–4, 5–9, …, 65–69), five 5-year time periods and eighteen overlapping 10-year birth cohorts (1905, 1910, …, 1990).

### RESULTS

During the study period 1969–1993, 23 289 ovarian cancer cases were identified in the provinces of British Columbia, Ontario and Saskatchewan. Serous tumours accounted for 24.2% ($n$ = 5645),

© Cancer Research Campaign 1999

**Table 1** Age-standardized incidence (per 100 000 women) and average annual percentage change (AAPC) by ovarian cancer histology-specific subtype, Ontario, British Columbia and Saskatchewan, 1969–1993

| Ovarian cancer histology subtypes | Incidence 1969–1971 | Incidence 1991–1993 | AAPC (%) 1969–1993 |
|---|---|---|---|
| All ovarian cancer | 14.56 | 13.71 | –0.5[a] |
| Serous | 1.45 | 4.61 | +5.4[a] |
| Mucinous | 1.24 | 1.72 | +0.71 |
| Endometrioid | 0.18 | 1.28 | +8.4[a] |
| Clear cell | 0.08 | 0.60 | +11.2[a] |
| Sex cord-stromal | 0.40 | 0.16 | –3.9[a] |
| Germ cell | 0.21 | 0.47 | +2.0[b] |
| Other classified epithelial | 9.82 | 3.57 | –4.8[a] |
| NOS/unclassified | 1.19 | 1.29 | –0.2 |

[a]$P < 0.01$; [b]$P < 0.05$.

**Table 2** Best log-linear models and model fit for ovarian cancer incidence by histology-specific subtype

| Ovarian cancer histology subtypes | Log-linear model[a] | df | Model fit deviance | *P*-value |
|---|---|---|---|---|
| All ovarian cancer | Age-cohort | 33 | 30.36 | 0.60 |
| Serous | Age-period-cohort | 30 | 29.78 | 0.50 |
| Mucinous | Age | 48 | 64.42 | 0.06 |
| Endometrioid | Age-period-cohort | 30 | 33.54 | 0.30 |
| Clear cell | Age-period | 43 | 54.02 | 0.12 |
| Sex cord-stromal | Age-drift | 47 | 59.78 | 0.10 |
| Germ cell | Age-cohort | 42 | 53.61 | 0.11 |
| Other classified epithelial | Age-period-cohort | 30 | 40.94 | 0.09 |
| NOS/unclassified | Age-period | 44 | 70.48 | 0.01 |

[a]Best model for each histology subtype

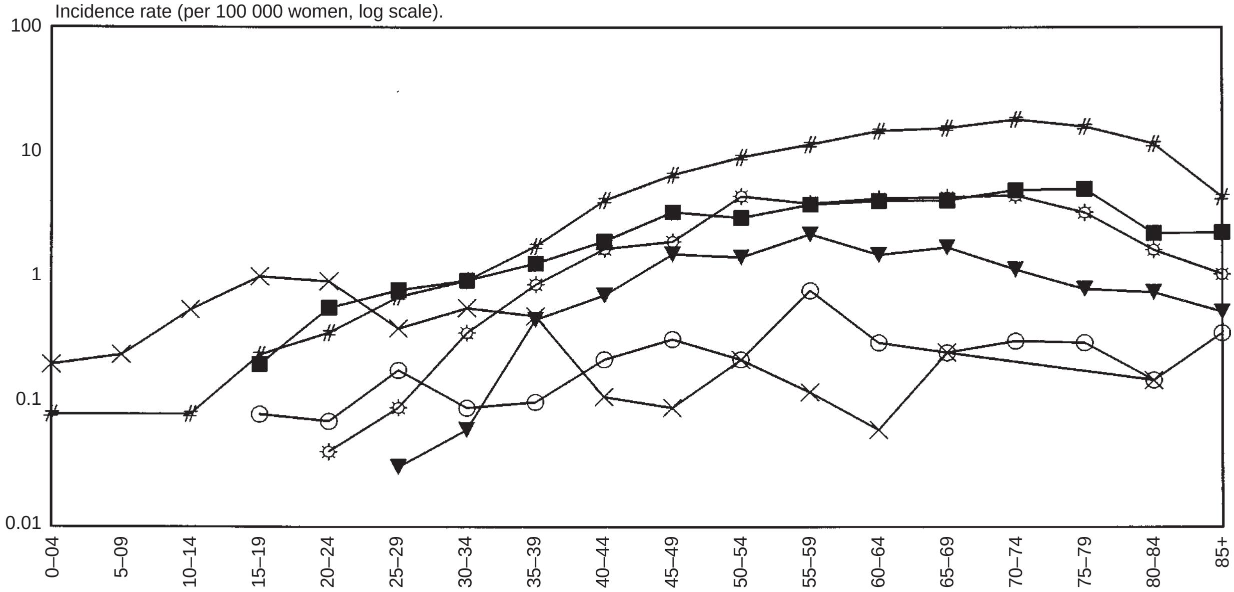

**Figure 1** Age-specific incidence rates of ovarian cancer histology subtypes among women in BC, ON and SASK, 1989–1993. #, serous; ■, mucinous; ☼, endometrioid; ▼, clear cell; ○, sex-cord; ×, germ cell followed by mucinous tumours 11.0% ($n$ = 2565), endometrioid tumours 7.1% ($n$ = 1650), clear cell tumours 2.4% ($n$ = 552), germ cell tumours 2.3% ($n$ = 546) and sex cord-stromal 1.8% ($n$ = 409). In addition, other classified epithelial ovarian tumours accounted for 39.8% ($n$ = 9258) and NOS/unclassified tumours 11.4% ($n$ = 2664) of all ovarian cancer cases.

ASIRs and AAPC for ovarian cancer histology subtypes for the period 1969–1993 are displayed in Table 1. The overall incidence rate of ovarian cancer dropped slightly over the time period. However, incidence rates according to histology subtypes showed different patterns. On one hand, a substantial increase in incidence rates was seen for serous tumours, endometrioid tumours, clear cell tumours and germ cell tumours, with an average annual percentage change of 5.4%, 8.4%, 11.2% and 2.0% respectively. On the other hand, incidence rates of sex cord-stromal and other classified epithelial ovarian tumours declined considerably with an average annual percentage change of 3.9% and 4.8%. No significant increase or decrease in incidence rates was observed for mucinous and NOS/unclassified tumours during the study period ($P > 0.05$).

The age-specific incidence rates of ovarian cancer overall increased exponentially with age, peaking at ages 75–84 (53.97 per 100 000 women) and decreasing slightly after age 85. Figure 1 displays the age-specific incidence rates by histology subtype for the period 1989–1993. Incidence rates of serous, mucinous and endometrioid tumours rose with age, peaking at 70–74 for serous tumours (18.53) and endometrioid tumours (4.57), and at 75–79 for mucinous tumours (5.14). A similar age-specific incidence pattern was seen for clear cell tumours and sex cord-stromal tumours. The incidence rate increased quickly after middle-age and peaked at ages of 55–59 for clear cell (2.24) and sex cord-stromal tumours (0.79). However, a different age-specific

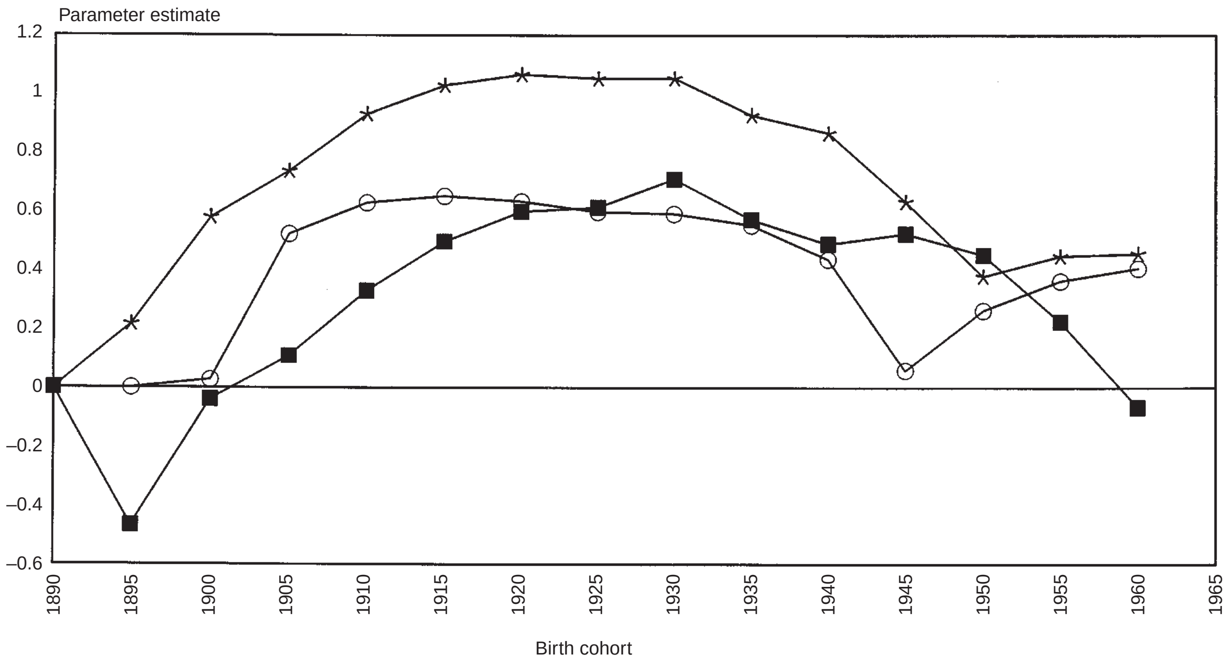

**Figure 2** Significant cohort effects on incidence trends of epithelial ovarian tumours among women in BC, ON and SASK, 1969–1993. No significant cohort effects were observed for mucinous and clear cell tumours. ■, serous; ○, endometrioid; ✳, other epithelial

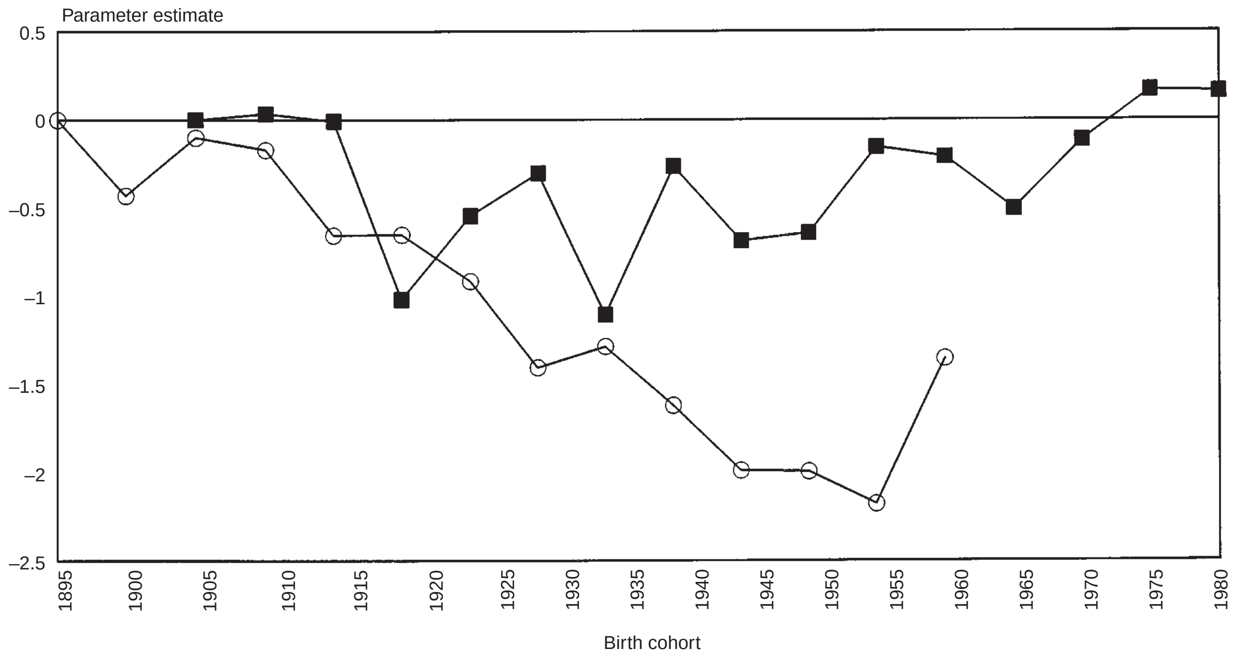

**Figure 3** Significant cohort effects on incidence trends of non-epithelial ovarian tumours among women in BC, ON and SASK, 1969–1993. ■, germ cell; ○, sex cord-stromal incidence pattern was observed for germ cell tumours. The incidence rate was generally high among children, peaking at age 15–19 (1.02) and declining thereafter.

Table 2 presents the best models for each subtype according to model fit and log-likelihood test. Age was found to have a significant impact on each of the ovarian cancer histology-specific incidence time trends. However, cohort and period have significant impacts on some histology subtypes but not on others.

A strong cohort effect was observed with the incidence trend of ovarian cancer overall but no significant period effect was

*© Cancer Research Campaign 1999*

detected. A rapidly increasing overall risk of ovarian cancer was seen among birth cohorts from 1890 to 1920. The risk, however, decreased consistently among women in the birth cohorts from 1925 to 1950 and appeared to level off in cohorts from 1955 to 1960. No cohort effect was observed for the incidence trend of NOS/unclassified tumours. The incidence trend of this subtype appears to be directly affected by the period effect.

Neither cohort effect nor period effect was detected in the incidence rates of mucinous tumours over the study period. In contrast, both cohort and period effect had significant impacts on the increasing incidence rates of serous and endometrioid tumours and decreasing incidence rates of other classified epithelial ovarian tumours. Figure 2 displays the significant cohort effects for epithelial ovarian tumours. An elevated risk of serous tumours among birth cohorts of 1895–1930 was observed. The risk decreased slightly among cohorts of 1930–1945 and dropped quickly in the younger cohorts of 1950–1960. The risk of endometrioid tumours appeared to be relatively stable between cohorts of 1905 and 1935; the risk dropped among cohorts of 1935–1940, but increased in younger cohorts 1950–1960. The cohort effect on the incidence trend of other classified epithelial ovarian tumours is the same as for ovarian cancer overall. However, the cohort effect on the incidence trend of clear cell tumours is not statistically significant.

The increase in incidence rates of serous and endometrioid tumours can also be partially explained by increasing period effects between 1969 and 1988 (data not shown) in addition to the cohort effect. Similarly, the significant decrease in incidence rates of other classified epithelial ovarian tumours is also influenced by a consistent decreasing period effect (data not shown). However, the dramatic increase in incidence rates of clear cell tumours appeared to be explained by an increasing period effect alone.

The increase in incidence rates of germ cell tumours was mainly influenced by cohort effect (Figure 3). In spite of variation due to the small number of cancer cases, a constant and significant increasing risk of germ cell tumours was observed in younger cohorts of 1965–1980. The declining incidence rate of sex cord-stromal ovarian cancer can be explained by both cohort effect and period effect. The risk dropped constantly for birth cohorts from the 1910 birth cohort to the 1950 cohort and increased slightly in the 1960 cohort (Figure 3). A consistent decreasing period effect over the study period was also evident (data not shown).

### DISCUSSION

Age-period-cohort analysis allows us to characterize the impacts of age, period and cohort on incidence time trends. However, limitations regarding this statistical methodology have been reported and interpretation of results from age-period-cohort analyses should be made with caution (Clayton and Schifflers, 1987*b*; Tarone and Chu, 1996). First of all, misclassification bias may be involved due to the notable overlap of birth cohorts. Second, interpretation of an age-period model requires taking into account the possibility that a period effect may occur when a change in exposure to a carcinogen affects all age groups equally. Third, the interpretation of an age-cohort model needs to consider the likelihood that a cohort effect may arise when there is a strong interaction between exposure and age group even if all age group exposed in a particular period were affected.

The relative completeness of histology-specific data in the three provincial cancer registries over the 25-year period provides a unique opportunity to examine histology-specific incidence trends of ovarian cancer. In our data, however, the proportions of serous, endometrioid and clear cell tumours were lower and the proportions of NOS/unclassified (11.4%) and other classified epithelial ovarian tumours (39.8%) were higher than those reported elsewhere (Koper et al, 1996; Bjorge et al, 1997), suggesting potential data quality concerns. The proportion of NOS/unclassified ovarian tumours remained about 11% over the study period; however, the proportion of all other classified epithelial ovarian tumours dropped sharply from approximately 60% to 23% during the study period. Ovarian cancer can be difficult to diagnose: in particular, determination of histology-specific subtypes is considerably inconsistent among pathologists. In addition, the criteria for histology classification of ovarian tumours changed over the study period. Thus diagnostic bias may be involved in this study, although the impact of this bias on the cohort effects estimated in this study is unclear. The histology-specific incidence rates for serous, endometrioid, clear cell tumours might be underestimated and the rates for all other epithelial ovarian tumours might be overestimated for the early study period. In age-period-cohort analysis, the changes of histology classification criteria over the study period would be mainly reflected in the period effect.

Over the study period 1969–1993, the age-standardized incidence rates for overall ovarian cancer dropped slightly. However, the rates of serous, endometrioid, clear cell and germ cell tumours increased significantly and the rates of sex cord-stromal and other classified epithelial ovarian tumours decreased considerably. The rates of mucinous and NOS/unclassified tumours remained unchanged. A direct comparison cannot be made because few epidemiological studies have reported histology-specific incidence trends for ovarian cancer (Cramer et al, 1981; Farrow and Rosenblatt, 1996).

The age-specific incidence rates by histology subtype observed in this study showed patterns similar to those reported in previous studies based on data from the US Third National Cancer Survey (Weiss et al, 1977), the Danish Cancer Registry (Ewertz et al, 1988) and the Vaud Cancer Registry in Switzerland (Levi et al, 1993).

### Period effect

For ovarian cancer overall, no period effect on the incidence time trend was observed, indicating no significant changes in cancer registry data capture procedures, no significant improvement in cancer diagnosis methods and no screening tests introduced during the study period. However, substantial increasing period effects were seen for serous, endometrioid and clear cell tumours and a dramatic decreasing period effect was observed for all other classified epithelial ovarian tumours. Decreasing period effects were also seen for sex cord-stromal and NOS/unclassified ovarian tumours. These opposite period impacts suggest changes in ovarian cancer histology diagnosis criteria. For example, improvements of histology classification criteria for serous, endometrioid and clear cell tumours may have resulted in decreasing rates of other classified epithelial ovarian tumours. The histology classification appears to remain unchanged for mucinous and germ cell tumours as no period effects were evident for these two subtypes.

### Cohort effect

Previous studies have suggested that epithelial and non-epithelial ovarian tumours have different aetiologies (Horn-Ross et al, 1992;

*© Cancer Research Campaign 1999*

Albrektsen et al, 1997). In addition, the aetiology of mucinous tumours is reported to be different from other epithelial tumours (Risch et al, 1996). An observation of significant cohort effects for incidence trends of serous and endometrioid tumours but not for trend of mucinous tumours appears to support Risch's hypothesis.

The cohort impact on incidence trends of serous and all other epithelial ovarian tumours may be closely associated with child-bearing patterns. For example, the increasing risk for birth cohorts around the turn of the century may be related to a substantial decline in parity among grandmothers of babies born in 'boom years' (Whittemore et al, 1992). Other factors such as increased fat intake and talc use may also contribute to the increasing risk (Risch et al, 1994b; Chang and Risch, 1997). On the other hand, the slightly decreasing risk in the 1930–1945 cohorts may be related to an increasing parity among mothers of the babies born in boom years. Data from Statistics Canada (1993) show that the crude birth rate (per 1000 population) decreased consistently from 45.0 to 20.6 in the period of 1860–1939 and the rate increased from 20.6 in 1939 to 28.2 in 1957 and stayed relatively high until 1964.

Oral contraceptive use and hysterectomy/tubal ligation may also play a role in the cohort effects observed for serous and endometrioid tumours. OC use and a history of hysterectomy/tubal ligation reduces the risk of ovarian cancer by half (Hankinson et al, 1993; Rosenberg et al, 1994). These two factors may be responsible for the decreasing risk of serous tumours among the younger cohorts of 1950–1960. Although data on OC use before 1977 are not available, data obtained from Saskatchewan Drug Plan (Saskatchewan Health Department, 1998) show a constant high prevalence of OC use among young women aged 15–29 (35%) from 1977 to 1992. The apparent increasing risk of endometrioid tumours among young cohorts of 1950–1960 is harder to explain. Hysterectomy and tubal ligations are reported to have specific strong protective effects for endometrioid tumours (Rosenblatt and Thomas, 1996). We found that incidence rates of hysterectomy and tubal ligations in Canada have declined since 1971 (Zhang, 1998), which may have influenced the slightly elevated risk of endometrioid tumours. To confirm this hypothesis, more studies are needed.

A few studies have investigated aetiologies of germ cell tumours (Walker et al, 1988; Horn-Ross et al, 1992). Since most germ cell tumours occur among girls and young women, causal factors may occur early in life, possibly even in utero. Similarly, a significant increasing risk of testicular germ cell tumours among boys and young men is reported in Canada (Liu et al, 1998). Perinatal factors including mother's exposure to exogenous oestrogen during the pregnancy and high pregnancy body weight have been associated with an increased risk. In addition, use of oral contraceptives is associated with an increased risk of germ cell tumours, but the role of parity is unclear (Horn-Ross et al, 1992; Adami et al, 1994; Albrektsen et al, 1997). Our study suggests that young women born in recent decades have experienced a higher risk of developing germ cell ovarian cancer. Further studies to examine the associations between hormonal exposure during pregnancy and OC use and the risk of developing germ cell ovarian tumours are warranted.

The aetiology of sex cord-stromal ovarian tumours is poorly understood. The evidence regarding the effect of parity on sex cord-stromal risk is controversial (Horn-Ross et al, 1992; Adami et al, 1994; Albrektsen et al, 1997). However, a history of using oral contraceptives or hormone replacement therapy (HRT) was associated with a decreased risk of developing sex cord-stromal ovarian tumours and a history of hysterectomy or tubal ligation did not affect the risk (Horn-Ross et al, 1992). In our study, we observed that women born in the 1910–1955 cohorts had a constant decreasing risk of developing sex cord-stromal ovarian tumours. This finding may be related to an increasing prevalence of HRT among Canadian women aged 45–59 from 15% in 1977 to 25% in 1996 (Saskatchewan Health Department, 1998). The hypothesis that OC use or HRT may reduce the risk of developing sex cord-stromal ovarian tumours needs to be confirmed in analytic epidemiological studies.

In summary, the different cohort patterns by histology subtype suggest that ovarian cancer is not a single disease and involves different aetiologies. In particular, the aetiologies of mucinous, germ cell and sex cord-stromal tumours are different and poorly understood; studies are warranted in this area. Further epidemiological studies examining ovarian cancer histology-specific incidence rates should also take into consideration the changes in classification criteria.

### ACKNOWLEDGEMENTS

The authors wish to acknowledge Ian MacNeill, consultant with the Cancer Bureau and Suzana Fraser of the Bureau of Surveillance and Field Epidemiology, Laboratory Centre for Disease Control, Health Canada, for their meaningful comments.

### REFERENCES

Adami HO, Hsieh CC, Lambe M, Trichopoulos D, Leon D, Persson I, Ekbom A and Janson PO (1994) Parity, age at first child, and risk of ovarian cancer. *Lancet* **344**:1250–1254

Adami HO, Bergstrom R, Persson I and Sparen P (1990) The incidence of ovarian cancer in Sweden, 1960–1984. *Am J Epidemiol* **132**: 446–452

Albrektsen G, Heuch I and Kvale G (1997) Full-term pregnancies and incidence of ovarian cancer of stromal and germ cell origin: a Norwegian prospective study. *Br J Cancer* **75**: 767–770

Amos CI and Struewing JP (1993) Genetic epidemiology of epithelial ovarian cancer. *Cancer* **71**: 566–572

Berg JW (1982) Morphologic classification of human cancer. In: *Cancer Epidemiology and Prevention,* 1st Edn, Schottenfeld D and Fraumeni J (eds), pp. 74–89. Oxford University Press: New York

Bjorge T, Engeland A, Hansen S and Trope CG (1997) Trends in the incidence of ovarian cancer and borderline tumours in Norway, 1954–1993. *Int J Cancer* **71**: 780–786

Chang S and Risch HA (1997) Perineal talc exposure and risk of ovarian carcinoma. *Cancer* **79**: 2396–2401

Clayton D and Schifflers E (1987*a*) Models for temporal variation in cancer rates, I. Age-period and age-cohort models. *Stat Med* **6**: 449–467

Clayton D and Schifflers E (1987*b*) Models for temporal variation in cancer rates, II. Age-period-cohort models. *Stat Med* **6**: 468–481

Cramer DW, Devesa SS and Welch WR (1981) Trends in the incidence of endometrioid and clear cell cancers of the ovary in the United States. *Am J Epidemiol* **114**: 201–208

Dos Santos Silva I and Swerdlow AJ (1995) Recent trends in incidence of and mortality from breast, ovarian and endometrial cancers in England and Wales and their relation to changing fertility and oral contraceptive use. *Br J Cancer* **72**: 485–492

Ewertz M and Kjaer SK (1988) Ovarian cancer incidence and mortality in Denmark, 1943–1982. *Int J Cancer* **42**: 690–696

Farrow DC and Rosenblatt KA (1996) Ovarian Cancer. In: *Cancer Epidemiology and Prevention*, 2nd Edn, Schottenfeld D and Fraumeni J (eds). Oxford University Press: New York

Farrow DC, Weiss NS and Lyon JL (1989) Association of obesity and ovarian cancer in a case-control study. *Am J Epidemiol* **129**: 1300–1304

Hankinson SE, Hunter DJ, Colditz GA, Willet WC, Stampfer MJ, Rosner B, Henneken CH and Speizer FE (1993) Tubal ligation, hysterectomy and risk of ovarian cancer: a prospective study. *JAMA* **270**: 2813–2818

© Cancer Research Campaign 1999 

Health and Welfare Canada (1993) *The Making of the Canadian Cancer Registry*: *Cancer Incidence in Canada and its Regions, 1969 to 1988*, pp. 10–19. Catalogue number C52-42/1992: Ottawa, Canada

Hempling RE, Wong C, Piver MS, Natarajan N and Mettlin CJ (1997) Hormone replacement therapy as a risk factor to epithelial ovarian cancer: results of a case-control study. *Obstet Gynecol* **89**: 1012–1016

Horn-Ross PL, Whittemore AS, Harris R, Itnyre J and the Collaborative Ovarian Cancer Group (1992) Characteristics relating to ovarian cancer risk: collaborative analysis of 12 US case-control studies. VI. Non-epithelial cancer among adults. *Epidemiology* **3**: 490–495

Koper NP, Kiemeney L, Massuger L, Thomas C, Schijf C and Verbeek A (1996) Ovarian cancer incidence (1989–1991) and mortality (1954–1993) in the Netherlands. *Obstet Gynecol* **88**: 387–393

Levi F, Franceschi S, La Vecchia C, Ruzicka J, Gloor E and Randimbison L (1993) Epidemiologic pathology of ovarian cancer from the Vaud Cancer Registry, Switzerland. *Ann Oncol* **4**: 289–294

Liu S, Wen SW, Mao Y, Mery L, Rouleau J (1998) Birth cohort effects underlying the increasing testicular cancer incidence in Canada. *Can J Public Health* (in press)

Mosgaard BJ, Lidegaard O, Kiaer SK, Schou G and Andersen AN (1997) Infertility, fertility drugs, and invasive ovarian cancer: a case-control study. *Fertil Steril* **67**: 1005–1012

National Cancer Institute of Canada (NCIC) (1998) *Canadian Cancer Statistics 1998*. NCIC: Toronto, Canada

Negri E, Franceschi S, Tzonou A (1991) Pooled analysis of 3 European case-control studies. I. Reproductive factors and risk of epithelial ovarian cancer. *Int J Cancer* **49**: 50–56

On L and Semenciw R (1995) *Disease Surveillance Sub-system*. Environmental Risk Assessment and Case Surveillance Division, Cancer Bureau, Health Canada: Ottawa, Canada

Risch HA, Marrett LD and How GR (1994*a*) Parity, contraception, infertility, and the risk of epithelial ovarian cancer. *Am J Epidemiol* **140**: 585–597

Risch HA, Jain M, Marrett LD and Howe GR (1994*b*) Dietary fat intake and risk of epithelial ovarian cancer. *J Natl Cancer Inst* **86**: 1409–1415

Risch HA, Marrett LD, Jain M and Howe GR (1996) Differences in risk factors for epithelial ovarian cancer by histologic type: results of a case-control study. *Am J Epidemiol* **144**: 363–372

Rosenberg L, Palmer JR, Zauber AG, Strom BL, Harlap S and Shapiro S (1994) A case-control study of oral contraceptive use and invasive epithelial ovarian cancer. *Am J Epidemiol* **139**: 654–661

Rosenblatt KA and Thomas DB (1996) Reduced risk of ovarian cancer in women with a tubal ligation or hysterectomy: The World Health Organization Collaborative Study of Neoplasia and Steroid Contraceptives. *Cancer Epidemiol Biomarkers Prev* **5**: 993–995

SAS Institute Inc. (1996) *SAS/STAT Software*: *Changes and Enhancements, Release 611*, pp. 383–490. SAS Institute Inc.: Cary, NC

Saskatchewan Health Department (1998) Utilization of contraceptives and hormone replacement therapy by Saskatchewan residents. Unpublished data. Saskatchewan Drug Plan and Extended Benefits. Regina, Saskatchewan, 1998.

Siskind V, Green A, Bain C and Purdie D (1997) Breastfeeding, menopause, and epithelial ovarian cancer. *Epidemiology* **8**: 188–191

Tarone RE and Chu KC (1996) Evaluation of birth cohort patterns in population disease rates. *Am J Epidemiol* **143**: 85–91

Walker AH, Ross RK and Haile RWC (1988) Hormonal factors and risk of ovarian germ cell cancer in young women. *Br J Cancer* **57**: 418–422

Weiss NS, Homonchuk T and Young J (1977) Incidence of the histologic types of ovarian cancer: the US Third National Cancer Survey, 1969–1971. *Gynecol Oncol* **5**: 161–167

Whittemore AS, Wu ML and Paffenbarger RS Jr (1988) Personal and environmental characteristics related to epithelial ovarian cancer. II. Exposures to talcum power, tobacco, alcohol, and coffee. *Am J Epidemiol* **128**: 1228–1240

Whittemore AS, Harris R, Itnyre J and the Collaborative Ovarian Cancer Group (1992) Characteristics relating to ovarian cancer risk: Collaborative analysis of 12 US case-control studies. II. Invasive epithelial ovarian cancers in white women. *Am J Epidemiol* **136**: 1184–1203

World Health Organization (1977) *International Classification of Diseases*, 1975 Revision, Vols 1 and 2. WHO: Geneva

World Health Organization (1976) *International Classification of Diseases for Oncology*. WHO: Geneva

Zhang J (1998) *Incidence Rates for Hysterectomy and Tubal Ligation in Canada and Provinces, 1970–1993*. Unpublished data, Cancer Bureau, Health Canada: Ottawa, Canada

 *© Cancer Research Campaign 1999*
